# Dr. Weatherill's Successful Case of Excision of the Cervix Uteri

**Published:** 1831

**Authors:** 


### LXVIII.

### DR. WEATHERILL'S SUCCESSFUL CASE OF EXCISION OF THE CERVIX UTERI.

IN the ninth volume of this series (No. XVIII, p. 518.) our readers will find an abbreviated account of a *successful* case of amputation of the cervix uteri, performed by Dr. Weatherill, of Liverpool, and published in a contemporary journal, about the middle of the year 1828. In that case, it is said that the cavity of the abdomen was laid open, and that the intestines rushed out; but that nevertheless, the woman perfectly recovered, and went to America cured. We made some severe animadversions on this case at the time; but as the patient had gone beyond the Western wave, we never expected to hear anything more of her. The re-publication of this journal in America has been the means of exciting the attention of our transatlantic brethren to the case in question, and it was only a few days ago, that we received a letter from Dr. Charles Hildreth, of Boston, Massachusetts, from which we make the following extract, for the benefit of Dr. Weatherill and those who place implicit credence in the *successful cures* performed by desperate operations in this and other countries.

To DR. JAMES JOHNSON.

"Sir,—You are so well, and so honourably known to the medical profession throughout the United States, that I feel myself, as it were, acquainted with you personally, and therefore I hesitate not to address you on the present occasion.

In the Medico-Chirurgical Review, for October 1828, page 518, you have noticed the case of "Successful Excision of the Cervix and Os Uteri," by Dr. Weatherill, of Liverpool. The patient therein mentioned died under my care, while physician to the Boston Infirmary, and of the disease for which she was operated on, not quite ten months previously by Dr. W. Particular circumstances prevented me from examining the body after death; but the poor woman experienced the most dreadful tortures, for which she was obliged to take enormous quantities of laudanum. The patient's name was Charlotte Lewin, aged 37, and recently arrived from Liverpool. Her narrative corresponded with Dr. W.'s account of the operation, but had any other than an Englishman stated the particulars of such a horrid procedure, we would not have believed it on this side of the Atlantic. The husband of this unfortunate female asserted that the bladder was wounded in the operation, and that the urine was never afterwards retained. For the truth of this, however, I cannot vouch, with certainty, as the body was not examined after death.

With sentiments of very high respect,
I am, Sir,
Your most obedient Servant,
CHARLES F. HILDRETH, M.D."

*Boston, Massachusetts,*
*April 9th*, 1831.

We do not feel inclined to make any new comments on Dr. Weatherill's operation. If the grave could give up faithful records of the dead, many similar *confirmations of successful operations* would, no doubt, be brought to light! The want of such records has probably done more to disturb the progress of medical and surgical knowledge, and to injure society at large, than many grievances more loudly complained of.

